# Evaluation of the novel algorithm of flexible ligand docking with moveable target-protein atoms

**DOI:** 10.1016/j.csbj.2017.02.004

**Published:** 2017-03-03

**Authors:** Alexey V. Sulimov, Dmitry A. Zheltkov, Igor V. Oferkin, Danil C. Kutov, Ekaterina V. Katkova, Eugene E. Tyrtyshnikov, Vladimir B. Sulimov

**Affiliations:** a Dimonta, Ltd, Nagornaya Street 15, Bldg. 8, Moscow 117186, Russia; b Research Computer Center, Moscow State University, Leninskie Gory 1, Bldg. 4, Moscow 119992, Russia; c Faculty of Computational Mathematics and Cybernetics of Lomonosov Moscow State University, Leninskie Gory 1, Bldg. 52, Moscow 119992, Russia; d Institute of Numerical Mathematics of Russian Academy of Sciences, Gubkin Street 8, Moscow, 119333, Russia

**Keywords:** Docking, Tensor train, Protein-ligand complex, Protein moveable atoms, Flexible ligand, Drug design

## Abstract

We present the novel docking algorithm based on the Tensor Train decomposition and the TT-Cross global optimization. The algorithm is applied to the docking problem with flexible ligand and moveable protein atoms. The energy of the protein-ligand complex is calculated in the frame of the MMFF94 force field in vacuum. The grid of precalculated energy potentials of probe ligand atoms in the field of the target protein atoms is not used. The energy of the protein-ligand complex for any given configuration is computed directly with the MMFF94 force field without any fitting parameters. The conformation space of the system coordinates is formed by translations and rotations of the ligand as a whole, by the ligand torsions and also by Cartesian coordinates of the selected target protein atoms. Mobility of protein and ligand atoms is taken into account in the docking process simultaneously and equally. The algorithm is realized in the novel parallel docking SOL-P program and results of its performance for a set of 30 protein-ligand complexes are presented. Dependence of the docking positioning accuracy is investigated as a function of parameters of the docking algorithm and the number of protein moveable atoms. It is shown that mobility of the protein atoms improves docking positioning accuracy. The SOL-P program is able to perform docking of a flexible ligand into the active site of the target protein with several dozens of protein moveable atoms: the native crystallized ligand pose is correctly found as the global energy minimum in the search space with 157 dimensions using 4700 CPU ∗ h at the Lomonosov supercomputer.

## 1 Introduction

The initial stage of new drug development is a search of the molecules which are inhibitors of a given target protein. Inhibitors block the active site of the protein associated with a disease and the disease is cured. A quick and effective solution of this problem decreases considerably material costs and duration of the whole drug development process. Nowadays, this problem can be addressed effectively with the help of computer simulations [1], [2]. Reliable predictions of the target protein inhibition by a low molecular weight ligand are defined by the accuracy of the docking programs. Docking programs carry out positioning of the ligand in the active site of the protein and calculate the protein-ligand binding free energy. The accuracies of positioning and the binding energy calculation are closely linked: faulty positioning cannot result in the high accuracy of the binding energy calculation based on the found ligand poses. The positioning accuracy of many existing docking programs is satisfactory and unpredictable positioning failures take place rather rarely. However, the accuracy of binding energy calculations for a randomly selected target protein is too bad: for the effective development of new inhibitors this accuracy should be better than 1 kcal/mol [3]. High accuracy of the protein-ligand binding energy calculations with docking programs is the key problem that should be solved in order to increase considerably effectiveness of the use of molecular modeling for the new inhibitors' development. This accuracy depends on many factors: the force field choice for modeling intra- and inter-molecular interactions, the solvent model, target protein and ligand models, the docking algorithm, the free energy calculation method, respective approximations and computer resources required for docking of one ligand.

In the frame of the docking procedure the protein-ligand binding energy Δ*G*_*bind*_ should be calculated as the difference between the free energy of the protein-ligand complex *G*_*PL*_ and the sum of free energies of the unbound protein *G*_*P*_ and the unbound ligand *G*_*L*_:

$$\Delta G_{\mathrm{bind}}=G_{\mathrm{PL}}-G_{P}-G_{L}.$$

Free energies of the protein, the ligand and their complex are described by respective energy landscapes and they can be calculated through the configuration integrals over the respective phase space. In the thermodynamic equilibrium the molecular system occupies its low energy minima. The configuration integral will come to the sum of configuration integrals over the separate low energy minima if these minima are separated by sufficiently high energy barriers [4], [5]. Thus, the docking accuracy is defined by the completeness of finding the low energy minima and by the accuracy of the configuration integral calculation in each of these minima.

The target protein model defines complexity and the volume of calculations and in many docking programs the rigid protein approximation is adopted. Moreover, in some docking programs, e.g. AutoDock [6], [7], ICM [8], DOCK [9], SOL [10], the grid of preliminary calculated potentials of the ligand probe atoms Coulomb and van der Waals interactions with the target protein is used in the main docking procedure. This results in the increase of computing speed but at the expense of restrictions on the docking performance and of worsening of the accuracy of binding energy calculations. The protein model with the preliminary calculated grid of potentials has a number of limitations. Firstly, this approach obviously cannot take into account mobility of the protein atoms. Secondly, such approach makes impossible carrying out the local optimization of the protein-ligand energy with the variation of coordinates of ligand and protein atoms. Thirdly, the local potentials in the grid nodes cannot represent the non-locality of the interaction of solute atom charges with polarized charges induced on the solvent excluded surface in implicit solvent models; as a result the interaction of the protein and the ligand with water cannot be treated accurately. Finally, the ligand poses found in this docking approach do not correspond to any energy minima because the local optimization of the energy is not performed.

Some time ago we decided to reject the docking procedure with preliminary calculated energy grid in the attempt to increase the accuracy of the protein-ligand binding energy calculations. Docking without the preliminary calculated energy grid requires much more computational resources even for vacuum calculations since one has to find low energy minima on the complicated multi-dimensional energy surface computing the energy in the frame of the whole given force field for each system conformation appearing in the minima search algorithm. Such docking programs, FLM [5] and SOL-T [11], have been developed for the rigid target protein and the flexible ligand. The parallel FLM program can perform the comprehensive minima search either in vacuum or with the rigorous implicit solvent model [12], [13]. However FLM requires too large supercomputer resources and it can be used mainly for finding low energy reference minima of protein-ligand complexes for the validation of docking algorithms [11] and force fields [5], [14]. The parallel SOL-T program employs the novel tensor train global optimization algorithm and it requires much less supercomputer resources than FLM. The docking positioning accuracy of FLM and SOL-T in vacuum for the rigid protein is comparable with one another at least for some test complexes [11]. The TT-docking algorithm was compared [15] with the genetic algorithm realized in the SOL program [10] with one and the same energy function on the preliminary calculated energy grid for rigid proteins and flexible ligands. In this case the ability to find the global energy minimum and the native (crystallized) position is close but the TT-docking algorithms perform about 10 times faster [15]. Further, it is demonstrated [5] that the ligand positioning accuracy is much better when the force field is used with a continuum solvent model. The ligand positioning accuracy is much better when the recent quantum chemical semiempirical methods, PM7 [14] and PM6 [16], are used instead of classical force fields.

However, proteins are flexible and dynamic molecular systems. A noticeable difference between protein's unbound (*apo*) and bound (*holo*) structures is sometimes observed. Ligand binding may cause a small side-chain rearrangement or individual atom's motions as well as significant conformational changes connected with domain motions. Thus, the protein flexibility can have a major impact on the molecular modeling results. It is reasonable to assume that the protein flexibility can significantly improve the docking positioning accuracy as well as the accuracy of the protein-ligand binding energy calculation on the base of docking results.

There are several methods to take protein flexibility into account [17], [18], [19], [20].

Soft docking [21] is the simplest method of protein flexibility accounting. It simulates the mobility of protein atoms by reducing the steric components of the scoring function (“softening” of van der Waals potentials). However, this approach can increase the number of false positives [22].

The ensemble docking approach is the docking into the ensemble of receptor conformations instead of docking into a single one. This method is popular because there is no need to change the existing docking algorithms in order to take protein flexibility into consideration. Multiple conformations are generated before docking and can be obtained from X-ray crystallography, of NMR spectroscopy or can be produced by molecular modeling, e.g. molecular dynamics. Moreover, ensemble docking can be carried out sequentially into each protein structure (“multiple-run” docking) [18], [23] or into one averaged structure [24] or into the dynamic pharmacophore model [25] (“single-run” docking). The composite structure also can be created on the basis of the ensemble of conformations and it consists of different parts of the original ensemble. Such composite structures are generated directly during the docking process [26], [27].

In the case of selective methods a few “critical” atoms or amino acid residues can move explicitly to explore the protein flexibility. Certain side chains of the active site are often chosen as the protein's degrees of freedom. Variation of their positions can be performed due to rotations around torsional degrees of freedom. Such rotation can be either continuous [28], [29] or discrete when the angles of rotation are determined on the basis of well-known libraries of rotamers [30], [31]. Selective methods also include the approach when only hydrogen atoms' reorientation is performed to optimize hydrogen bonds between protein and ligand [32], [33]. Some implementations of selective methods vary the protein conformations after the ligand optimization in the rigid protein [34]. There is also an approach that allows optimization of both the ligand and the side chains of protein simultaneously. However, this can be done only for a strongly restricted number (no more than 22) of protein and ligand degrees of freedom [35].

Protein flexibility can also be investigated in the context of post-docking (“induced-fit” methods): first, the ligand position is found using rigid docking or soft docking, and then the additional optimization of the protein conformation is performed using a selective approach [36]. Sometimes this post-optimization can be performed by the Monte Carlo method or molecular dynamics [37], [38] to take into account flexibility of the whole protein. A more refined docking approach combines multiple local optimizations with the subsequent global optimization in vicinities of picked out local minima [39]. Initially, 1000 local minima were found with the help of the energy gradient optimization with variations of coordinates of ligand and protein atoms (more than 1000 atoms) using randomly selected initial poses of the ligand in the active site of the target proteins. Then the global optimization by the Monte Carlo method was performed in the close vicinity of most perspective local minima [39].

There is also the Monte Carlo docking procedure [40] where random target protein side-chain perturbations are followed by the local energy optimization with variations of coordinates of ligand and protein atoms and this procedure is repeated iteratively. The docking method of “molecular relaxations” [41] employs the molecular dynamics (MD) approach, but this method is supplanted now by more accurate and more computationally expensive MD methods of the protein-ligand binding energy calculation, e.g. the free energy perturbation procedure [42].

Algorithms of most modern docking programs are based on the docking paradigm [5], [11], [14]. This paradigm assumes that the ligand binding pose in the active site of the target protein corresponds to the global minimum of the protein-ligand energy function or is near it. Due to this paradigm the docking problem is reduced to the search of the global minimum on the multi-dimensional protein-ligand energy surface. The dimensionality of this surface (*d*) is defined by the number of protein-ligand system degrees of freedom. Docking of small molecules into the rigid target protein is reliable when the number of ligand degrees of freedom (translations and rotations as a whole and torsions) is not more than 20–25 [10]. For larger dimensionality of the search space, i.e. for larger number of protein-ligand system degrees of freedom, commonly used docking algorithms, e.g. the genetic algorithm, are not able to perform docking successfully. Therefore inclusion of coordinates of moveable protein atoms into the docking procedure increases significantly the dimensionality of the global minimum search space and the solution of the docking problem requires more effective global optimization algorithms.

Is it possible to perform the global optimization of the protein-ligand energy considering the ligand flexibility and the mobility of protein atoms simultaneously and equally at least for several dozens of protein moveable atoms? The present study demonstrates that the answer is positive: yes, it is possible to perform successfully such docking employing the novel tensor train global optimization algorithm [11]. In this study we describe the main features of this novel algorithm, the respective program SOL-P for docking flexible ligands into target proteins with moveable atoms [43] and the results of validation of the ligand positioning accuracy for a test set of 30 protein-ligand complexes [11]. However, the protein-ligand binding energy calculation is out of the scope of this work. It is demonstrated here that even limited mobility of protein atoms results in considerable improvement of the docking positioning accuracy. Whilst the present results were obtained for the MMFF94 force field [44] in vacuum, the performance of SOL-P allows including in the docking procedure one of either rigorous (PCM or COSMO) or heuristic (Generalized Born) solvent models [45]. The ability to perform docking with the PCM solvent model has been already demonstrated by the FLM program which demands more computing resources [5]. Although the SOL-P program does not outperform existing docking programs either in terms of positioning accuracy or speed of calculation, it opens the way for the accurate calculation of the protein-ligand binding free energy by employing the sets of low-energy minima of the molecular systems (the target protein, the ligand and their complex) which are carefully found for a given force field with a continuum solvent model. If low energy minima are found, the whole configuration integral defining the free energy of the respective molecular system can be accurately calculated as a sum of configuration integrals over these separated minima [4], [5]. Such an accurate approach cannot be handled by commercial, superfast software that runs on laptops in seconds.

## 2 Materials and methods

For the realization of the novel docking algorithm we use the MMFF94 force field [44] in vacuum. While looking for low-energy minima, ligands are considered to be fully flexible and some of protein atoms are moveable. The force field determines the energy of the protein-ligand complex for its every conformation. The MMFF94 force field combines sufficiently good parameterization based on ab initio quantum-chemical calculations of a broad spectrum of organic molecules and the well-defined procedure of atom typification applicable to an arbitrary organic compound. This force field is not worse than many other popular force fields such as: AMBER [46], [47], OPLS-AA [48], CHARMM [49] etc. MMFF94 is implemented in the SOL docking program [10] used successfully for new inhibitors' development [50], [51], [52]. Moreover, it has been recently shown that the docking paradigm is true for some protein-ligand complexes, if the energy of the complex is calculated in the frame of the MMFF94 force field in vacuum [5]. The docking paradigm is not satisfied for many complexes, if the energy is calculated with MMFF94 in vacuum [5], but accounting for solvent in the frame of an implicit solvent model improves the situation significantly [5]. However, it is found in [5] and later is supported in the quasi-docking procedure [14] that the recent quantum-chemical semiempirical PM7 method with solvent is much better than MMFF94 with solvent. The same finding is presented independently in [16] comparing the PM6-D3H4X semiempirical method with eight different force fields including AMBER [46], [47] and several empirical and knowledge-based force fields. Unfortunately, these quantum-chemical methods are much slower than force fields. Keeping all this in mind we investigate here the influence of protein atoms' mobility in the docking procedure on the quality of ligand positioning using only the MMFF94 field in vacuum. The results will be much better, if either MMFF94 is used with the solvent model or PM7 is used with the solvent model.

### 2.1 TT-docking

The novel docking algorithm (TT-docking) utilizes the TT global optimization method. It is based on the novel methods of tensor analysis. The detailed description of this algorithm is presented elsewhere [11], [15] and here we describe only its main features.

The Tensor Train decomposition for *d*-dimensional tensors was introduced to numerical analysis in 2009 [53] as a means to fight against the so-called *curse of dimensionality*, given by the fact that the number of entries of a *d*-dimensional tensor grows exponentially in *d* and can easily exceed the number of atoms in the universe even for a kind of “small sizes”, i.e. for *d* = 300 and 2 points at each dimension. Consequently, the list of entries cannot be used for practical computations. The Tensor Train (TT) is a decomposition in which the number of the tensor representation parameters grows in *d* just linearly. Moreover, despite some other classical decompositions (such as CPD — the Canonical Polyadic Decomposition [54]), the TT algorithms reduce all computations to structured low-rank matrices associated with the given tensor. In our optimization procedure this structure is used to navigate in the space for where to search for better minima. This procedure is essentially based on the TT Cross algorithm [55] that constructs a TT decomposition using only a small portion of the entries of the given tensor. Eventually the number of those entries used during the optimization depends on *d* just polynomially, and the *curse of dimensionality* mentioned above is no longer an obstacle.

The continuous protein-ligand energy function is transformed into the multi-dimensional array (tensor) and the novel tensor analysis methods are applied for the search of the tensor element with the maximal absolute value: obviously, the docking problem is the global minimization problem but it can be easily transformed to an equivalent problem of the magnitude maximization. If *d* is the number of degrees of freedom of the protein-ligand complex, then we can introduce the grid in the configuration space with *n*_*i*_ nodes in each direction *i* = 1 , 2 … *d*. If the grid is fine enough, then the solutions of continuous and discrete problems are expected to be close.

The basis of this consideration is the Tensor Train (TT) decomposition [53], [56] of a tensor $A\in R^{n_{1}\times \ldots \times n_{d}}$ in the form:

$$A\left(i_{1},\ldots ,i_{d}\right)=\sum_{\alpha_{1}=1,\ldots ,\alpha_{d-1}=1}^{r_{1},\ldots ,r_{d}}G_{1}\left(i_{1},\alpha_{1}\right)G_{2}\left(\alpha_{1},i_{2},\alpha_{2}\right)\ldots G_{d-1}\left(\alpha_{d-2},i_{d-1},\alpha_{d-1}\right)G_{d}\left(\alpha_{d-1},i_{d}\right)$$

The numbers *r*_1_ , … , *r*_*d* − 1_ are called TT-ranks of the tensor; for convenience, dummy ranks *r*_0_ ≡ *r*_*d*_ ≡ 1 are also introduced. The 3-dimensional tensors $G_{i}\in R^{r_{i-1}\times n_{i}\times r_{i}}$ are called cores or carriages of the tensor train. If TT-ranks are reasonably small, then the TT decomposition possesses several very useful properties [53], [56]. However, we cannot afford computing or storing all the elements for large tensors. Therefore, it becomes crucial to have for tensors a fast approximation method utilizing only a small number of their elements. Such a method was proposed and called the TT-Cross method [55]. It heavily exploits the matrix cross interpolation [57], [58], [59], [60], [61] algorithm applied cleverly, although heuristically, to selected submatrices in the unfolding matrices of the given tensor. The matrix $A_{k}\in R^{n^{k}\times n^{d-k}}$, *A*_*k*_(*i*_1_ … *i*_*k*_, *i*_*k* + 1_ … *i*_*d*_) = *A*(*i*_1_, *i*_2_, … , *i*_*d*_) is called the *k*-th unfolding matrix of the tensor *A*. Such matrices are highly connected with TT-decomposition, TT-rank *r*_*k*_ is just the rank of the matrix *A*_*k*_.

The TT-Cross method iteratively improves the sets of interpolation points searching for submatrices of larger volume (determinant in modulus) and consequently the elements of larger magnitude. This property allows one to take it as a base for the global optimization method [11].

The TT-docking iteratively performs the following steps:

1. Generation of submatrices of unfolding matrices using sets of tensor elements.
2. Interpolation of submatrices using TT-Cross method with rank ≤* r*_max_.
3. A set of interpolation points for each submatrix contains elements with large values in modulus.
4. Rough local optimization of interpolation points (protein-ligand poses) by the simplex method, addition of optimized point projections to the tensor and to the interpolation point sets.
5. Updating of each set of interpolation points of the unfolding matrix by merging the interpolation points of the previous unfolding matrix and ones of the subsequent unfolding matrix.
6. Addition of the best points (ligand poses) to the interpolation point set of the unfolding matrix, and transition to step 1 using the obtained point set as the tensor elements.

The complexity of the TT global optimization method is *O*(*dnr*_max_^2^) functional evaluations, *O*(*dr*_max_) local optimizations and *O*(*dnr*_max_^3^) arithmetic operations, where *r*_*max*_ is the maximal rank of the Tensor Train decomposition, *n* is the initial grid size along one dimension and *d* is the number of dimensions. It is easy to see that operations for different unfolding matrices could be performed independently, and we need synchronization only when constructing the new points at the end of each iteration. Moreover, a parallel implementation of the matrix cross method is also available [62]. In the result, we have a parallel version of the TT global optimization algorithm with parallel complexity *O*(*r*_max_) functional evaluations, *O*(1) local optimizations and *O*(*d* + *r*_max_^2^) arithmetic operations.

### 2.2 SOL-P docking program

The parallel SOL-P docking program is constructed on the base of the TT-docking algorithm (see above). The SOL-P program is developed for finding the low energy local minima spectrum of protein-ligand complexes, proteins or ligands including the respective global energy minimum. The energy of each molecule conformation is calculated directly in the frame of the MMFF94 force field [44] in vacuum without any simplification or fitting parameters. The conformation space of the system coordinates is formed by translations and rotations of the ligand as a whole, by the ligand torsions and also by Cartesian coordinates of the selected target protein atoms. The description of the ligand flexibility with torsions is used as a basic approach in many docking programs (AutoDock [6], [7], ICM [8], DOCK [9], SOL [10] and GOLD [63]) to decrease the dimensionality of the search space. Certainly, in this approach some features of the ligand flexibility, e.g. the macrocyclic system flexibility, are not taken into account. The flexibility of the target protein is described here by the variations of Cartesian coordinates of the protein atoms located near the ligand atoms for certain ligand poses. This is the first step to the approach of the protein flexibility and it is chosen here only for the uniformity of consideration of different proteins and ligands and to keep restricted the change of the initial protein configuration taken from Protein Data Bank (PDB) [64]. While solving a particular docking problem for a given target protein it is better to choose moveable protein atoms more cleverly, by sampling configurations of whole groups of the covalently bound protein atoms, such as side chains or loops, selected on the base of a priori knowledge. But such detailed investigation is out of the scope of the present work. The parallel MPI (message passing interface) based SOL-P program is written on C ++ with usage of BLAS and LAPACK libraries. Main SOL-P parameters are: the maximal rank *r*_max_ of the TT-Cross approximation method, the power *m* of the discretization degree of the search space (the initial grid size is equal to *n* = 2^*m*^ along one dimension) and the number of iterations of the TT global optimization algorithm. The initial grid is introduced in the *d*-dimensional search space to transform the continuous global optimization problem to the discrete one: finding the maximal in magnitude element of the *d*-dimensional tensor. Each point in the search space corresponds to a certain pose of the ligand in a certain configuration of the active site of the target protein and each element of the *d*-dimensional tensor corresponds to the MMFF94 energy of the protein-ligand complex in a given node of the grid. The total number of nodes in the grid (2^*md*^) is made large enough (see Section 2.6) to keep smoothness of the continuous MMFF94 energy function in the discrete problem: energy values in neighboring nodes are close to one another. Moreover, it is convenient to apply the TT magnitude maximization to the functional *f*(*x*, *E*_⁎_) = exp{100arccot[*E*(*x*) − *E*_⁎_]}, where *E*(*x*) is the dimensionless MMFF94 energy for the given configuration *x* of the protein-ligand complex, *E*_⁎_ is the currently found global minimum. This function transforms the minimization problem to the maximization one. This function also zeroes large positive MMFF94 energy values arising due to the van der Waals repulsion of closely located atoms and it better separates low energy minima. As it is mentioned in the previous section there is a rough local energy optimization in the TT-docking algorithm by the Nelder-Mead simplex method [65] within the Subplex algorithm [66] implemented as Sbplx program in NLOpt library [67].

### 2.3 Moveable atoms

The ligand is considered as flexible with variations of its torsions, and also some protein atoms are moveable. In the present consideration a protein atom is moveable when it is close to at least one of reference ligand poses. The protein atom is close to a ligand pose when the distance between this protein atom and at least one ligand atom is less than a given threshold. In one extreme case, only the nonoptimized native (crystallized) ligand pose can be included into the set of reference ligand poses. In another extreme case, the reference poses of the ligand can be taken from the set of ligand poses corresponding to low-energy minima of the protein-ligand system which were found by SOL-P for the flexible ligand and the rigid protein. In this case the maximal number of protein atoms will be moveable. In the present work we took three ligand poses as reference ones: the ligand pose corresponding to the global protein-ligand energy minimum found by the FLM program [11] for the rigid protein, the locally optimized native ligand pose and the nonoptimized native ligand pose. None of movements of whole side chains is considered in this study. Such choice of the reference ligand poses is taken here only for the uniformity of the consideration of all different proteins and ligands of the test set. Determination of moveable protein atoms is carried out by our original specially written program Mark-PMA (Mark Protein Moveable Atoms) with the MLT (Moveable Layer Thickness) parameter defining the threshold distance. The MLT parameter is taken up to 3 Å in the present investigation.

### 2.4 Docking procedure

**Fig. 1:**
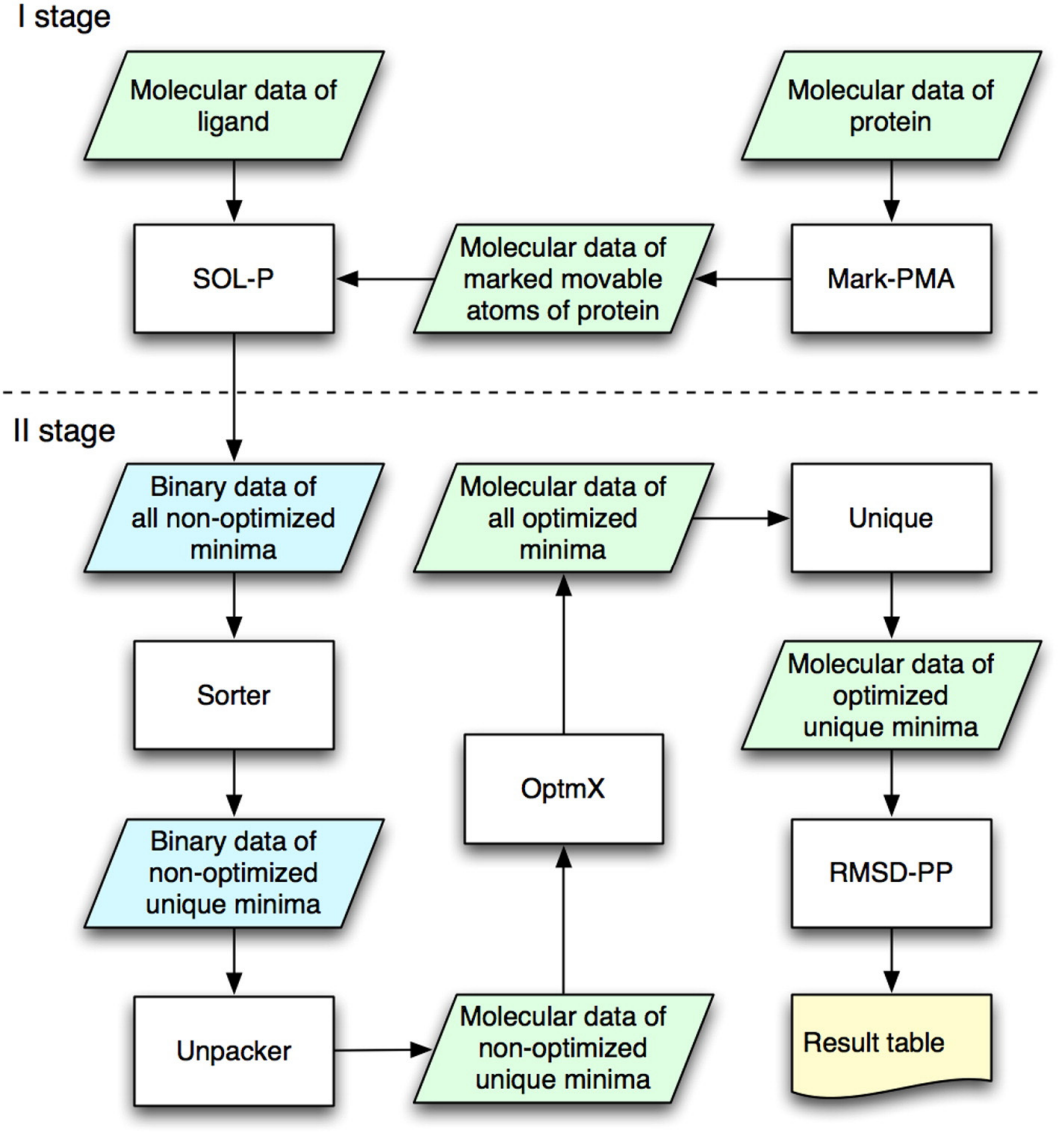
Flowgraph of the program complex for low energy local minima search with flexible ligand and moveable target protein atoms. Stage I: the data preparation and TT global energy minima search with the SOL-P program. Stage II: the analysis of binary data with the “non-optimized minima” obtained from the SOL-P program and preparation of the table with the results and the final minima set.

The molecular data of the ligand and the protein with the marked moveable atoms are the input of the SOL-P program (shown in I stage in Fig. 1). The SOL-P program uses a cube centered in the geometrical center of the native ligand position in the crystallized protein-ligand complex as the spatial region for the low-energy minima search: all found ligand positions have their geometrical centers inside this cube (the docking cube). The cube is aligned along the Cartesian axes of the protein-ligand system. Each of the moveable protein atoms can move inside its own small cube centered in the initial atom position taken from the crystallized protein-ligand complex. Geometrical characteristics of the big docking cube and small cubes of moveable protein atoms are specified in the parameter file of the SOL-P program. In this work we set the docking cube edge equal to 10 Å and the small cube edge equal to 1 Å. We restrict motions of the moveable protein atoms in such a way that their Cartesian coordinates can change in the range of ± 0.5 Å from their positions in the crystallized structure because even small changes can make big differences in the protein-ligand energetics [3]. The position and the size of the docking cube in the active site of the target protein are usually defined by binding sites which can be interesting from a pharmacological point of view. For the positioning accuracy validation in this study we choose the center of the docking cube in the geometrical center of the native (crystallized) ligand position of the respective complex. Such choice of the docking cube implies that all low energy minima, which were found in the docking procedure, correspond to a single locus of the ligand binding. The SOL-P program performs MPI-parallelized search for the low-energy minima of protein-ligand complexes by the TT-docking algorithm containing the rough local optimization by the simplex method. The ligand has six rotational-translational degrees of freedom as a whole rigid body plus torsional degrees of freedom for each single non-cyclic bond; each of the protein moveable atoms has three degrees of freedom — its Cartesian coordinates. The optimized target function is the protein-ligand complex total energy calculated by the MMFF94 force field in vacuum without any simplification or fitting parameters. Data about all found low-energy minima including protein-ligand configurations are too large to be saved in the molecular data format. These configurations are saved as the binary data (shown in Fig. 1 as “Binary data of all non-optimized minima”).

### 2.5 Analysis of local minima

At stage II in Fig. 1 the post-processing of low energy configurations stored in the binary data is performed with the Sorter program. The Sorter program sorts the “nonoptimized minima” by their MMFF94 energies in vacuum and excludes minima with similar ligand positions — only one minimum with the lowest energy is being kept. Two ligand positions are considered similar if RMSD between them is less than a given threshold (0.1 Å), where RMSD is calculated atom-to-atom without chemical symmetry accounting. Thus, all the remaining low-energy configurations (“unique non-optimized minima” in Fig. 1) have different ligand positions. Then, the Unpacker program performs exporting all unique low-energy configurations from the binary file to the file with molecular format MOL2. The post-processing of low energy protein-ligand configurations consists of the performance of two programs: OptmX and Unique (Fig. 1). The OptmX program locally optimizes all of the “unique non-optimized minima”. For these purposes, the OptmX program uses the L-BFGS algorithm [68], [69] applied to the local optimization of the MMFF94 energy function in vacuum with variations of Cartesian coordinates of all ligand atoms and moveable protein atoms. Optimization of different minima is MPI-parallelized. After this optimization, the “all optimized minima” (Fig. 1) set is obtained. However, many of these minima may become similar again. Therefore, we need to re-exclude similar minima. The Unique program excludes similar minima from the “all optimized minima” set as follows. Among several close configurations only the minimum with the lowest energy is being kept as it is made in the binary data file post-processing by the Sorter program. However, in contrast to the Sorter program the protein moveable atoms are also taken into account in RMSD calculation, and the RMSD is calculated with chemical symmetry analysis.

Analysis of the local minima remaining after post-processing is carried out by the RMSD-PP program which calculates RMSD (with respect to all ligand atoms) between the ligand pose in a certain energy minimum of the protein-ligand complex and the ligand pose in the energy minimum corresponding to the native ligand position obtained after the local optimization from its configuration in the crystallized complex. The RMSD here is calculated taking into account the approximate chemical symmetry analysis as follows. A special attribute (so called “chemical digest”; in the present implementation it is the 32-bit integer number) is assigned to each atom, depending only on the MMFF94 type of this atom and the MMFF94 types of the adjacent atoms bound with this atom by chemical bonds, as follows. The selected atoms, including the analyzed atom, are ordered to a sequence, where atom “A” precedes atom “B” if “A” is closer to the analyzed atom (i.e. number of separating bonds from the analyzed atom is less for “A”) or, in case of equally distanced “A” and “B”, if “A” has a lower MMFF94-type (an integer from 1 to 99). Then, this sequence of MMFF94-types is processed by a hash function; in the present implementation, we used the CRC32 (32-bit Cyclic Redundancy Check) algorithm [70]. The obtained hash function value is the “chemical digest”. The neighbors are analyzed by the breadth-first search [71] until the given depth (we set this parameter equal to 13) will be reached. So, chemically symmetric atoms have the same “chemical digest”. Unfortunately, not every one-to-one atom mapping, keeping the “chemical digest” invariant, can preserve the whole chemical structure. Nevertheless, the “chemical digest” heuristic can filter off many of the wrong atom-to-atom mapping during the RMSD calculation. After the “chemical digest” calculation, all atoms with the same “chemical digest” are grouped. Within the group, all possible squared distances are calculated, where the first atom position belongs to the first configuration and the second atom position belongs to the second configuration. Then, the atom-to-atom assignment is searched by the Hungarian method [72]. So, the calculated RMSD doesn't exceed (and in many cases equal to) the lowest possible RMSD with keeping the chemical structure atom-to-atom mapping. This RMSD with approximate chemical symmetry accounting is a good metric to estimate the geometrical difference between two configurations of a protein-ligand complex; it can correctly discard geometrical pseudo-differences such as phenyl residue flip, comparing to the native atom-to-atom RMSD calculation.

As a result the RMSD-PP program creates in its output (Fig. 1) the resulting table containing: the minimum index, the minimum energy, RMSD from the optimized native configuration and the distance from the ligand geometric center in the given minimum to the ligand geometric center in the optimized native configuration. The energy minima are sorted by their energy in the ascending order; that is, every minimum gets its own index equal to its number in this sorted list of minima. The lowest energy minimum has the index equal to 1.

Some minima from the list might be close in space to the optimized native ligand position. We designate the index of the minimum having RMSD from the optimized native ligand position less than 2 Å as “Index of the minimum Near Optimized Native” or “INON.” If there are several such minima which are close to the optimized native ligand position, we will choose the minimum with the lowest energy (with the lowest index) as “INON”. When INON = 1 the docking paradigm is satisfied: the global minimum of the protein-ligand energy is near the native configuration. If there are no minima with the ligand pose near the optimized native configuration among all minima found by the SOL-P program, we use notation INON = inf.

It is useful to enhance the requirement on the minimum situated near the optimized native ligand position including the restriction on its energy and to introduce another index (EN) as the energy index of the minimum being near the optimized native ligand in space (RMSD < 2 Å as it is used in the definition of INON) and in energy (in the energy interval ± 1 kcal/mol from the energy of the optimized native ligand). If there are several such minima, we will choose the minimum with the lowest energy (with the lowest index) as EN. Index EN demonstrates how far from the global minimum is the energy of the minimum found near the optimized native ligand pose. If EN is equal to a small positive integer, it means that the docking program finds a minimum near the optimized native ligand position and its energy is one of the lowest among the whole found minima spectrum. Index EN is useful when the energy of the optimized native ligand pose differs strongly from the energy of the global minimum.

In the present consideration we compare the energy minima found by the SOL-P program with ones obtained by the FLM program [11] with the same target function — energy in the frame of the MMFF94 force field in vacuum. FLM performs exhaustive search of low energy local minima of protein-ligand complexes in the rigid protein and flexible ligand approximation performing massive parallel energy minima search and employing large computing resources (about 20,000 CPU ∗ h per one complex) available at supercomputer Lomonosov of Moscow State University [73].

### 2.6 Optimal SOL-P parameters

To choose optimal parameters of the SOL-P program we execute two sets of test calculations. First, calculations for the selection of the optimal parameters of the TT global optimization method (TT-docking) are performed. Second, calculations for selection of the optimal number of the moveable protein atoms are carried out. The first set of test calculations are carried out for 7 different protein-ligand complexes with rigid proteins (they are shown in Table 1). TT-docking performance is investigated with different values of two parameters: the maximal rank *r*_max_ = {4, 8, 16} and the initial grid size *n* = {2^8^, 2^16^}.

Results of this testing demonstrate that for the higher initial grid size even the lowest tested maximal rank *r*_max_ = 4 is enough to find the optimum reliably and precisely. However, the increase of the initial grid size leads to slower convergence of the method and the iteration number must be larger (for *n* = 2^16^ from 10 to 15 iterations need to be performed). The high grid size for ranks 8 and 16 makes computations significantly slower, thus the initial grid size of 2^12^ is used for such ranks. For such initial grid size the computation time is reduced by 1.5 times and the number of iteration decreases. Finally, three sets of optimal parameters are chosen: the first set with *r*_max_ = 4 and *n* = 2^16^, the second set with *r*_max_ = 8 and *n* = 2^12^, and the third set with *r*_max_ = 16 and *n* = 2^12^. For all sets the same number of iterations equal to 15 is used.

Second testing calculations are carried out for 3 different complexes (Table 2) with different numbers of moveable protein atoms. The MARK-PMA program defines different numbers of moveable protein atoms for these complexes. Numbers of moveable protein atoms for respective complexes and the calculated values of INON index are presented in Table 2.

**Fig. 2:**
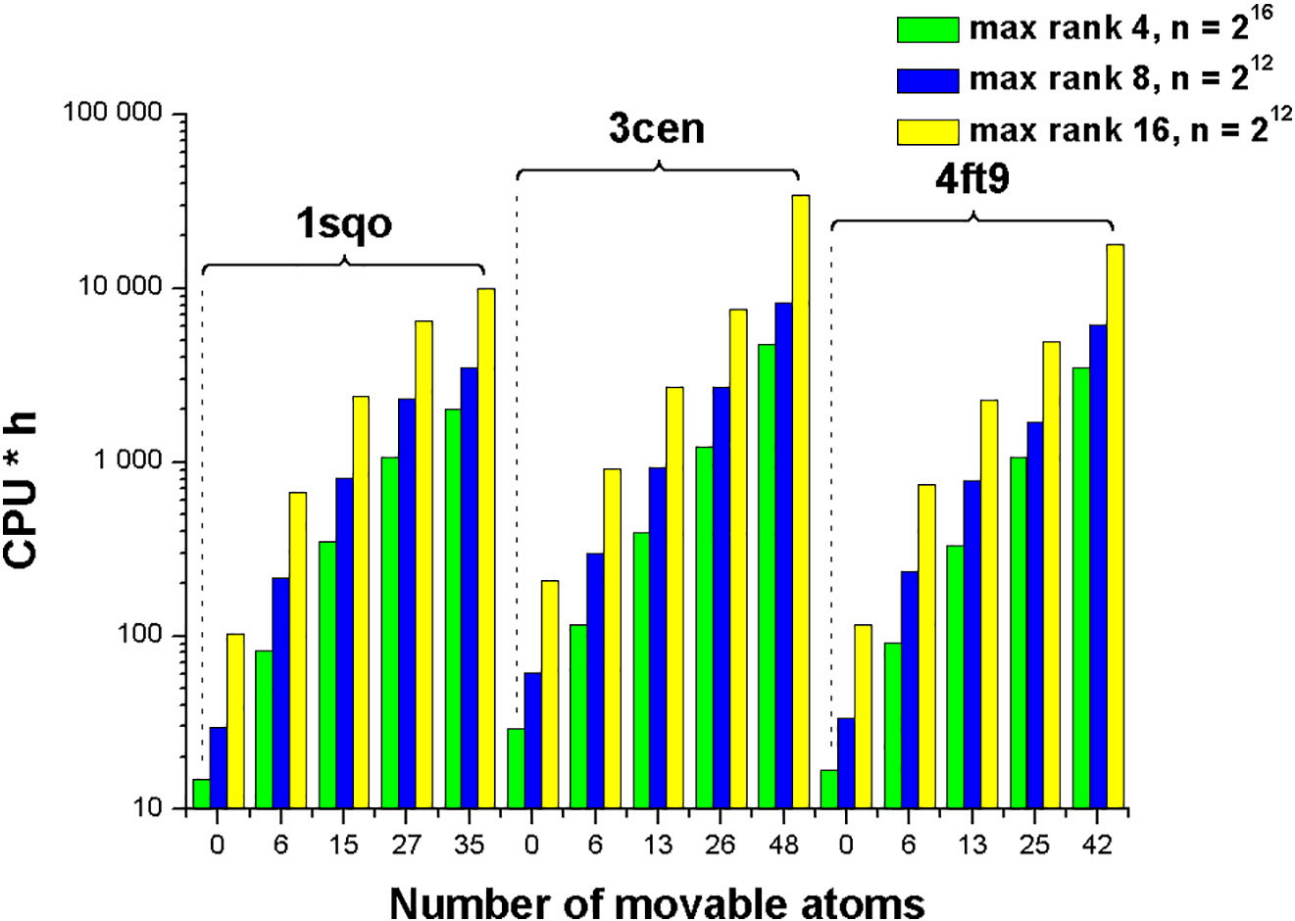
Dependence of computing resources on the number of protein moveable atoms for the native ligand docking by the SOL-P program with different sets of TT-docking parameters. Integer *n* is the initial grid size.

It can be seen that INON = 1 for all sets of protein moveable atoms for 1SQO complex. It means that the ligand pose corresponding to the global energy minimum is situated near the optimized native configuration and the docking paradigm is satisfied for the case of the rigid protein as well as for all selected cases of moveable protein atoms. For the 3CEN complex SOL-P does not find the energy minimum near the optimized native configuration for the rigid protein as well as for 6 protein moveable atoms. However, for 13, 26 and 48 protein moveable atoms INON is equal to 1 or 2 corresponding to cases when SOL-P finds the minimum near the optimized native configuration and its energy is lowest (its index is 1or 2) among energies of all other minima found by SOL-P. For 4FT9 complex SOL-P finds the minimum close to the native configuration (*INON* ≠ *inf*) but there are many minima with energies lower than energy of this close to the native configuration minimum. This means that the target energy function defined by the MMFF94 force field in vacuum is not adequate for this complex. Moreover, for 42 protein moveable atoms SOL-P cannot find the minimum close to the native configuration (INON = inf). Probably, in this case the TT-docking algorithm cannot find respective minima due to the high number of degrees of freedom for the given system: 137 = 126 (protein) + 11 (ligand). Strictly speaking the docking paradigm is not satisfied for the 4FT9 complex. So, we see that for some complexes (e.g. 1SQO) the docking paradigm is satisfied for the rigid protein as well as for the protein up to 35 moveable atoms. For some complexes (e.g. 3CEN) the docking paradigm is satisfied only for a sufficiently large number (13, 26, 48) of protein moveable atoms and SOL-P is able to find the global energy minimum in the search configuration space of 157 = 144 (protein) + 13 (ligand) degrees of freedom. For other complexes (e.g. 4FT9) the MMFF94 force field energy in vacuum is not adequate and the energy surface is so complicated that for the too large number of protein moveable atoms (42) SOL-P is not able to find minima near the native configuration. Computing resources needed for the native ligand docking using the SOL-P program with different TT-docking parameters and different numbers of protein moveable atoms are presented in Fig. 2.

Comparing computing resources in Fig. 2 and results of INON calculations in Table 2 two cases of optimal numbers of protein moveable atoms are chosen (13–18 and 25–35 atoms depending on the complex) in the present study for more broad validation.

### 2.7 Validation set of protein-ligand complexes

For low-energy local minima search we use 30 protein-ligand complexes with experimentally known 3D structures [11] (see Table 3). All protein-ligand complexes are chosen with good resolution from PDB [64]. The ligand variety covers a wide range from small and rigid ligands (e.g. the ligand of the 1C5Y complex) to big and flexible ones (e.g. the ligand of the 1VJ9 complex). For all these complexes the locally optimized ligand native position has RMSD from the original (crystallized) native pose less than 1.5 Å. Thus the locally optimized ligand native position still can represent the native ligand pose.

Protein structures are prepared as follows. All the records corresponding to atoms, ions and molecules which are not a part of the protein structure are eliminated from the PDB files of the complexes. Hydrogen atoms are added to this structure by the APLITE program [10]. The APLITE program adds hydrogen atoms according to the standard amino acid protonation states at pH = 7 and performs the protein energy optimization with variations of positions of all hydrogen atoms in the frame of the MMFF94 force field keeping fixed all protein heavy atoms. Ligands are also taken from PDB files. Hydrogen atoms are added to ligands by the Avogadro program [74].

As can be seen from Table 3 the largest part of all protein moveable atoms are hydrogen ones almost for all test complexes. Movements of hydrogen atoms during the docking process can be favorable for the hydrogen bond formation. However, we do not consider that properties of the MMFF94 force field [44] enable SOL-P to reproduce hydrogen bonds with high precision and there is no sense to analyze their formation in the present study.

## 3 Results

The total number of low energy minima found by the SOL-P program, *N*_*tot*_, for each complex varies considerably for different complexes depending on the complexity of the protein-ligand energy surface. This number can be as small as *N*_*tot*_ = 25 for the rigid protein of the 1C5Y complex and it can be as large as *N*_*tot*_ = 7149 for 1VJ9 with 30 protein moveable atoms. The *N*_*tot*_ number expands with the increase of the number of protein moveable atoms for any tested complex when the dimensionality of minima search space increases, e.g. for the 1I7Z complex *N*_*tot*_ = 362 for the rigid protein and *N*_*tot*_ = 1437 for 29 moveable protein atoms. Values of *N*_*tot*_ found by SOL-P and FLM are comparable for many complexes.

Computing resources for all 30 test protein-ligand complexes are 5–120, 110–1300 and 600–3200 CPU ∗ h for the rigid proteins, for the cases of 13–18 and 25–35 protein moveable atoms, respectively. So, docking with 13–18 protein moveable atoms needs dozens of times more computing resources as compared with the rigid protein case and docking with 25–35 protein moveable atoms needs several times more resources as against docking with 13–18 protein moveable atoms.

A priori there is one special local energy minimum in the protein-ligand energy minima spectrum for any energy function calculated in the frame of any force field either in vacuum or in solvent. It is the minimum obtained by the local optimization of the protein-ligand energy beginning from the ligand pose in the crystallized protein-ligand complex. The ligand pose in this local minimum we call optimized native ligand pose. The local energy optimization is performed with variations of either only ligand atoms or ligand and moveable protein atoms. Due to the docking paradigm this local minimum must be in the low energy part of the whole energy minima spectrum and the docking program must find it. The ability to find this energy minimum is one of indicators of the high quality of the low energy minima search algorithm: finding this minimum is the necessary condition of the thoroughness of the docking program performance. The SOL-P program finds such minimum for 10, 14 and 13 complexes (out of 30 complexes) for docking into the rigid protein, into the protein with 13–18 moveable atoms and 25–35 moveable atoms, respectively. We see that moveable protein atoms improve the ability of the SOL-P program to find the optimized native ligand pose. However, this feature of the SOL-P program is worse than one of the FLM programs which finds the optimized native ligand pose for 17 complexes of the same test set performing the exhaustive low energy minima search [11]. For 7 complexes (1C5Y, 1I7Z, 1O3P, 2PAX, 3PAX, 4FSW and 4FT0) both SOL-P for rigid proteins and for proteins with moveable atoms and FLM (for rigid proteins) find the optimized native ligand minimum. For 3 complexes (4FTA, 1SQO and 1EFY) SOL-P can find and FLM cannot find the optimized native ligand minimum. For 10 complexes neither SOL-P (with and without protein moveable atoms) nor FLM (for the rigid proteins) can find the optimized native ligand minimum. This result shows that the low energy minima search by either SOL-P or FLM docking programs is not perfect for some complexes. These failures could be partly due to the non-adequate target energy function: search algorithms look for low energy minima and can miss the optimized native ligand pose if its energy is too high.

**Fig. 3:**
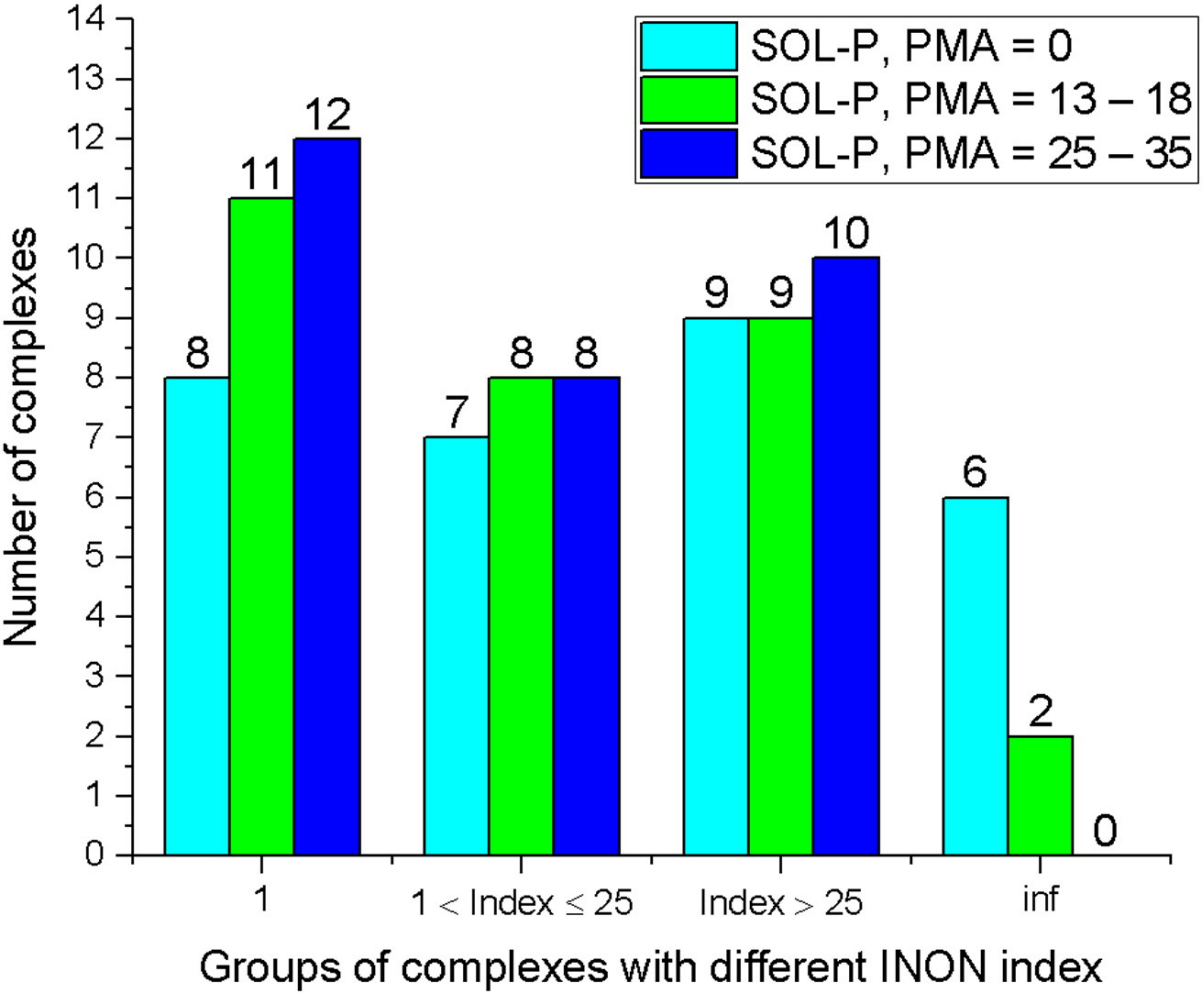
Numbers of complexes with different values of INON index. PMA indicates the range of protein moveable atoms for the SOL-P program. INON is the index of the minimum having RMSD from the optimized native ligand position less than 2 Å; if there are several such minima, the minimum with the lowest energy (with the lowest index) should be taken.

The validation shows that SOL-P finds either the global minimum or one of low energy minima corresponding to the ligand pose being near the optimized native ligand pose for the rigid protein and/or for the protein with moveable atoms for more than two thirds of the whole test set of protein-ligand complexes (for 22 out 30) (see Table 4): for these 22 complexes INON = 1 or INON ≤ 25 and the docking paradigm is fulfilled for them in the frame of the MMFF94 force field in vacuum. The test complexes are collected in groups in respect with values of their INON index in Fig. 3. This assertion is true also for FLM performance for the rigid proteins practically for the same complexes (see Table 4).

Taking into account protein atoms' mobility is crucial for 4 complexes (1J01, 1K1J, 1MQ6 and 3CEN) out of 30. SOL-P does not find any minima near the optimized native ligand pose for docking into the rigid protein (INON = inf). However, when mobility of protein atoms is taken into account, the docking procedure finds near the optimized native ligand pose either the global minimum (INON = 1) or one of the lowest energy minima (INON ≤ 25). Moreover, SOL-P with 25–35 protein moveable atoms always finds energy minima corresponding to the ligand pose near the optimized native ligand pose. On the other hand, for rigid proteins SOL-P and FLM cannot find such minima (INON = inf) for 6 and 5 complexes, respectively. It is worth to note that SOL-P is able to find the minimum near the optimized native ligand pose for all 5 complexes where FLM is not able to do this.

The FLM program (performing the exhaustive minima search) finds the global energy minimum near the optimized native ligand pose (INON = 1) for 13 complexes: 1C5Y, 1F5L, 1HPV, 1I7Z, 1J01, 1LQD, 1PPC, 1SQO, 1VJ9, 2PAX, 3CEN, 3KIV and 3PAX. The SOL-P program (with and without protein moveable atoms) finds also the global energy minimum near the optimized native ligand pose (INON = 1) for almost all these complexes except only two complexes: 1VJ9 and 1PPC.

Further, SOL-P finds not more than 10 minima near the optimized native ligand pose for most of the test complexes and only for few complexes the number of such minima is 11–36. Moreover, some of such minima are global energy minima (INON = 1) and their energies are close to the energies of respective optimized native ligand minima (EN = 1) for 5 or 6 complexes depending on mobility of protein atoms, e.g. for complexes 1C5Y, 1I7Z, 1J01, 1LQD, 1SQO and 2PAX with moveable protein atoms (see Table 4). There are 8 such minima found by the FLM program.

Therefore, we can say that in tote the SOL-P program (with and without protein moveable atoms) works not worse than the FLM program and much faster than the latter.

Our observation that neither SOL-P nor FLM can find any minimum near the optimized native ligand pose for 11 complexes (out of 30) is connected with inadequacy of the energy target function calculated in the frame of the MMFF94 force field in vacuum. It has been previously demonstrated [5] that protein-ligand energy calculation in the frame of the MMFF94 force field in solvent (with an implicit model) improves docking performance of the FLM program for the rigid proteins and with such target energy function SOL-P should also work better.

## 4 Conclusions

The validation results of the novel supercomputer SOL-P docking program are presented. This program performs docking of a flexible ligand into the protein with moveable atoms on the base of the search of the low-energy minima spectrum of a protein-ligand complex. Protein and ligand atoms' mobility is taken into account simultaneously and equally in the docking procedure. During this search the energy of each configuration of the protein-ligand complex is computed directly with the MMFF94 force field without simplifications and any fitting parameters. The grid of precalculated energy potentials of probe ligand atoms in the field of target protein atoms is not used. For the docking positioning validation energies of low-energy minima and their spatial locations corresponding to the ligand poses are carefully analyzed. Low-energy minima spectra of 30 protein-ligand complexes are investigated in the frame of the MMFF94 force field in vacuum.

It is shown that the program is able to perform docking of a flexible ligand into the active site of the target protein taking mobility of assigned protein atoms into account: up to 157 degrees of freedom in the conformation space using about 9 h at 512 core of the Lomonosov supercomputer [73]. As far as we know this is the first time when the docking program is able to perform successfully the global energy minimum search in the conformational space with such a large dimensionality. This result is achieved due to the usage of the novel docking algorithms (TT-docking) which are based on the so-called Tensor Train decomposition of multi-dimensional arrays (tensors) and the TT global optimization method [11], [15]. TT-docking does not suffer from the curse of dimensionality. In principle the SOL-P program has no restrictions (except the availability of supercomputer resources) to perform docking with a larger number of moveable protein atoms including side-chain mobility and/or docking the very flexible ligands such as oligopeptides.

It is found that docking performance of the SOL-P program is comparable with one of the FLM program, which executes the exhaustive energy minima search for rigid target proteins due to employment of much larger computing resources. It is demonstrated that in some cases docking results are being improved even when small movements of protein atoms are taken into account in the docking procedure.

It is demonstrated that the docking paradigm is fulfilled for the target energy function calculated in the frame of the MMFF94 force field in vacuum for a flexible ligand and for target proteins with 25–35 moveable atoms for two thirds of the whole test set of protein-ligand complexes. Taking into account an implicit solvent model in the calculation of the energy of the protein-ligand complexes should improve the positioning performance of the SOL-P docking program as it is observed for the FLM program [5].

The SOL-P docking program can be used for finding spectra of low-energy minima of the protein, the ligand and their complex in the frame of a given force field, and these spectra can be used for the binding free energy calculation through the configuration integrals over separated minima of the respective systems. This approach should improve accuracy of the protein-ligand binding energy calculations and it is similar to the “mining minima” method [4]. However our approach differs from the “mining minima” method mainly by more uniform and exhaustive low-energy local minima search instead of the exploration of the configuration space along a combination of low-frequency modes as it is made by the “mining minima” method [4].

The present investigations became possible due to the computing resources of M.V. Lomonosov Moscow State University supercomputer Lomonosov [73].

## Abbreviations

TT: tensor train
PDB: protein data bank
NMR: nuclear magnetic resonance
MMFF94: Merck molecular force field
MPI: message passing interface
BLAS: basic linear algebra subprograms
LAPACK: linear algebra package
RMSD: root-mean-square deviation
INON: index of the minimum near the optimized native ligand position
EN: index of the minimum being near the optimized native ligand in space and in energy

## Figures and Tables

**Table 1:** Complexes for testing parameters of the TT-docking algorithm. PDB ID is the ID of the respective protein-ligand complex taken from Protein Data Bank [64].

Protein name	PDB ID	Number of ligand atoms including hydrogen ones	Number of ligand torsions
Urokinase	1C5Y	20	2
	1F5L	24	6
	1VJA	61	17
	1VJ9	74	19
CHK1 (checkpoint kinase 1)	4FTA	35	6
Thrombin	1TOM	64	10
ERK2 (extracellular signal-regulated kinase 2)	4FV6	57	12

**Table 2:** Values of INON index (Index of the minimum Near Optimized Native) for three protein-ligand complexes with different numbers of protein moveable atoms. PDB ID is the ID of the respective protein-ligand complex taken from Protein Data Bank [64].

PDB ID (number of ligand torsions)	Number of protein moveable atoms	INON		
		*r*_max_ = 4 *n* = 2^16^	*r*_max_ = 8 *n* = 2^12^	*r*_max_ = 16 *n* = 2^12^
1SQO (3)	0, 6, 15, 27, 35	1	1	1
3CEN (7)	0, 6	inf	inf	inf
	13	1	inf	17
	26	1	1	2
	48	2	2	1
4FT9 (5)	0	16	24	29
	6	17	21	21
	13	17	18	19
	25	15	15	15
	42	inf	inf	inf

**Table 3:** Validation set of protein-ligand complexes. Numbers of atoms includes hydrogen ones. *N*_*P*_ is the total number of the protein moveable atoms. *N*_*H*_ is the number of the protein moveable hydrogen atoms.

Protein name	PDB ID	Num. of ligand torsions	Numbers of ligand atoms	Numbers of moveable protein atoms 13–18 *N*_*P*_/*N*_*H*_	Numbers of moveable protein atoms 25–35 *N*_*P*_/*N*_*H*_
Urokinase	1C5Y	2	20	14/8	26/17
	1SQO	4	34	15/8	27/17
	1F5L	6	24	16/8	27/14
	1O3P	6	46	17/11	28/20
	1VJA	17	61	16/8	28/15
	1VJ9	19	74	16/9	30/18
CHK1 (checkpoint kinase 1)	4FSW	0	26	15/11	29/22
	4FT0	3	42	15/11	26/20
	4FT9	5	32	13/10	25/20
	4FTA	6	35	15/11	31/22
Factor Xa	1MQ6	7	54	14/10	30/18
	2P94	7	60	13/10	29/21
	3CEN	7	50	13/9	26/17
	1LQD	8	61	17/10	31/22
Poly(ADP-ribose) polymerase	2PAX	1	24	14/7	20/10
	1EFY	3	33	14/12	27/18
	3PAX	3	20	14/9	25/15
ERK2 (extracellular signal-regulated kinase 2)	4FV5	8	52	16/13	33/25
	4FV6	12	57	18/11	26/19
Thrombin	1TOM	10	64	14/6	25/15
	1DWC	12	71	16/12	26/19
Trypsin	1PPC	6	69	15/8	25/16
	1K1J	10	68	13/8	27/19
GNC92H2 antibody	1I7Z	5	44	17/16	29/26
Apolipoprotein	3KIV	6	22	13/5	30/10
Beta-1,4-xylanase	1J01	6	35	15/8	29/18
Ricin	1BR5	7	29	15/9	27/18
Neuraminidase	1B9V	10	50	16/12	33/23
Hen egg-white lysozyme	1LZG	11	56	15/10	26/19
HIV-1 protease	1HPV	14	70	14/9	31/21

**Table 4:** Indexes EN and INON for the SOL-P program with different numbers of protein moveable atoms (0, 13–18 and 25–35) and for the FLM program with rigid proteins.

Complex id	EN/INON, SOL-P 0	EN/INON, SOL-P 13–18	EN/INON, SOL-P 25–35	EN/INON, FLM
1B9V	inf/344	513/353	inf/333	inf/inf
1BR5	144/45	241/23	inf/29	inf/309
1C5Y	1/1	1/1	1/1	1/1
1DWC	inf/20	inf/289	inf/98	inf/377
1EFY	72/46	38/20	40/16	158/81
1F5L	1/1	2/1	2/1	1/1
1HPV	inf/1	6/1	2/1	98/1
1I7Z	1/1	1/1	1/1	1/1
1J01	inf/inf	1/1	1/1	1/1
1K1J	inf/inf	inf/inf	inf/19	1/4
1LQD	inf/5	1/1	1/1	1/1
1LZG	inf/inf	inf/1270	inf/771	inf/inf
1MQ6	inf/inf	2/2	inf/3	7/4
1O3P	13/11	13/2	2/1	16/14
1PPC	inf/inf	inf/26	inf/51	1/1
1SQO	1/1	1/1	1/1	1/1
1TOM	inf/181	inf/465	inf/570	inf/inf
1VJ9	inf/26	inf/29	inf/23	48/1
1VJA	inf/50	inf/inf	inf/127	41/4
2P94	inf/2	inf/2	27/2	36/2
2PAX	1/1	1/1	1/1	1/1
3CEN	inf/inf	inf/1	inf/1	94/1
3KIV	9/1	5/1	4/1	12/1
3PAX	2/1	2/1	2/1	2/1
4FSW	6/5	6/5	6/5	8/7
4FT0	21/20	20/15	15/9	32/30
4FT9	inf/23	35/21	40/22	46/29
4FTA	176/176	370/370	415/415	inf/inf
4FV5	inf/231	87/87	122/84	189/122
4FV6	inf/337	inf/213	inf/325	inf/inf

## References

[1] Sliwoski G., Kothiwale S., Meiler J., Lowe E.W. (2014). Computational methods in drug discovery. Pharmacol Rev.

[2] Sadovnichii V.A., Sulimov V.B., Voevodin V.V., Sadovnichii V.A., Savin G.I. (2009). Supercomputing technologies in medicine. Supercomputing technologies in science, education, and industry.

[3] Mobley D.L., Dill K.A. (2009). Binding of small-molecule ligands to proteins: “what you see” is not always “what you get”. Structure.

[4] Chen W., Gilson M.K., Webb S.P., Potter M.J. (2010). Modeling protein-ligand binding by mining minima. J Chem Theory Comput.

[5] Oferkin I.V., Katkova E.V., Sulimov A.V., Kutov D.C., Sobolev S.I., Voevodin V.V. (2015). Evaluation of docking target functions by the comprehensive investigation of protein-ligand energy minima. Adv Bioinforma.

[6] Morris G.M., Goodsell D.S., Halliday R.S., Huey R., Hart W.E., Belew R.K. (1998). Automated docking using a Lamarckian genetic algorithm and an empirical binding free energy function. J Comput Chem.

[7] Huey R., Morris G.M., Olson A.J., Goodsell D.S. (2007). A semiempirical free energy force field with charge-based desolvation. J Comput Chem.

[8] Neves M.A.C., Totrov M., Abagyan R. (2012). Docking and scoring with ICM: the benchmarking results and strategies for improvement. J Comput Aided Mol Des.

[9] Allen W.J., Balius T.E., Mukherjee S., Brozell S.R., Moustakas D.T., Lang P.T. (2014). DOCK 6: impact of new features and current docking performance. J Comput Chem.

[10] Sulimov A.V., Kutov D.C., Oferkin I.V., Katkova E.V., Sulimov V.B. (2013). Application of the docking program SOL for CSAR benchmark. J Chem Inf Model.

[11] Oferkin I.V., Zheltkov D.A., Tyrtyshnikov E.E., Sulimov A.V., Kutov D.C., Sulimov V.B. (2015). Evaluation of the docking algorithm based on tensor train global optimization. Bull South Ural State Univ Ser Math Model Program Comput Softw.

[12] Romanov A.N., Jabin S.N., Martynov Y.B., Sulimov A.V., Grigoriev F.V., Sulimov V.B. (2004). Surface generalized born method: a simple, fast and precise implicit solvent model beyond the Coulomb approximation. J Phys Chem A.

[13] Sulimov V.B., Mikhalev AYu, Oferkin I.V., Oseledets I.V., Sulimov A.V., Kutov D.C. (2015). Polarized continuum solvent model: considerable acceleration with the multicharge matrix approximation. Int J Appl Eng Res.

[14] Sulimov AV, Kutov DC, Katkova EV, Sulimov VB. Combined docking with classical force field and quantum chemical semiempirical method PM7. *Adv Bioinforma*. Accepted 22 December 2016. Volume 2017, Article ID 7167691, 6 pp., http://dx.doi.org/10.1155/2017/7167691. 10.1155/2017/7167691 PMC5278191 28191015

[15] Zheltkov D.A., Oferkin I.V., Katkova E.V., Sulimov A.V., Sulimov V.B., Tyrtyshnikov E.E. (2013). TTDock: docking method based on tensor train. Vychislitelnie Metody Programmirovanie (Numer Methods Program).

[16] Pecina A., Meier R., Fanfrlík J., Lepšík M., Řezáč J., Hobza P. (2016). The SQM/COSMO filter: reliable native pose identification based on the quantum-mechanical description of protein–ligand interactions and implicit COSMO solvation. Chem Commun.

[17] Antunes D.A., Devaurs D., Kavraki L.E. (Dec 2015). Understanding the challenges of protein flexibility in drug design. Expert Opin Drug Discovery.

[18] Fischer M., Coleman R.G., Fraser J.S., Shoichet B.K. (2014). Incorporation of protein flexibility and conformational energy penalties in docking screens to improve ligand discovery. http://www.nature.com/nchem/journal/v6/n7/full/nchem.1954.html.

[19] B-Rao C., Subramanian J., Sharma S.D. (2009). Managing protein flexibility in docking and its applications. http://www.sciencedirect.com/science/article/pii/S1359644609000063.

[20] Sousa S.F., Ribeiro A.J., Coimbra J.T., Neves R.P., Martins S.A., Moorthy N.S.H.N. (2013). Protein-ligand docking in the new millennium — a retrospective of 10 years in the field. Curr Med Chem.

[21] Jiang F., Kim S.H. (1991). “Soft docking”: matching of molecular surface cubes. J Mol Biol.

[22] Buonfiglio R., Recanatini M., Masetti M. (2015). Protein flexibility in drug discovery: from theory to computation. ChemMedChem.

[23] Martinez-Ramos F., Fonseca-Sabater Y., Soriano-Ursua M.A., Torres E., Rosales-Hernandez M.C., Trujillo-Ferrara J.G. (2013). *o*-Alkylselenenylated benzoic acid accesses several sites in serum albumin according to fluorescence studies, Raman spectroscopy and theoretical simulations. Protein Pept Lett.

[24] Osterberg F., Morris G.M., Sanner M.F., Olson A.J., Goodsell D.S. (2002). Automated docking to multiple target structures: incorporation of protein mobility and structural water heterogeneity in AutoDock. Proteins.

[25] Carlson H.A., Masukawa K.M., Rubins K., Bushman F.D., Jorgensen W.L., Lins R.D. (2000). Developing a dynamic pharmacophore model for HIV-1 integrase. J Med Chem.

[26] Claussen H., Buning C., Rarey M., Lengauer T. (2001). FlexE: efficient molecular docking considering protein structure variations. J Mol Biol.

[27] Corbeil C.R., Englebienne P., Moitessier N. (2007). Docking ligands into 11 flexible and solvated macromolecules. 1. Development and validation of FITTED 1.0. J Chem Inf Model.

[28] Abagyan R., Totrov M., Kuznetsov D. (1994). ICM—a new method for protein modeling and design: applications to docking and structure prediction from the distorted native conformation. J Comput Chem.

[29] Schnecke V., Kuhn L.A. (2000). Virtual screening with solvation and ligand induced complementarity. Perspect Drug Discovery Des.

[30] Leach A.R. (1994). Ligand docking to proteins with discrete side-chain flexibility. J Mol Biol.

[31] Ding F., Yin S., Dokholyan N.V. (2010). Rapid flexible docking using a stochastic rotamer library of ligands. J Chem Inf Model.

[32] Jones G., Willett P., Glen R.C. (1995). Molecular recognition of receptor sites using a genetic algorithm with a description of desolvation. J Mol Biol.

[33] Smiesko M. (2013). DOLINA — docking based on a local induced-fit algorithm: application toward small-molecule binding to nuclear receptors. J Chem Inf Model.

[34] Schnecke V., Swanson C.A., Getzoff E.D., Tainer J.A., Kuhn L.A. (1998). Screening a peptidyl database for potential ligands to proteins with side-chain flexibility. Proteins.

[35] Morris G.M., Huey R., Lindstrom W., Sanner M.F., Belew R.K., Goodsell D.S. (2009). AutoDock4 and AutoDockTools4: automated docking with selective receptor flexibility. J Comput Chem.

[36] Sherman W., Day T., Jacobson M.P., Friesner R.A., Farid R. (2006). Novel procedure for modeling ligand/receptor induced fit effects. J Med Chem.

[37] Sokkar P., Sathis V., Ramachandran M. (2012). Computational modeling on the recognition of the HRE motif by HIF-1: molecular docking and molecular dynamics studies. J Mol Model.

[38] Schaffer L., Verkhivker G.M. (1998). Predicting structural effects in HIV-1 protease mutant complexes with flexible ligand docking and protein side-chain optimization. Proteins.

[39] Apostolakis J., Pluckthun A., Caflisch A. (1998). Docking small ligands in flexible binding sites. J Comput Chem.

[40] Borrelli K.W., Cossins B., Guallar V. (2010). Exploring hierarchical refinement techniques for induced fit docking with protein and ligand flexibility. J Comput Chem.

[41] Tsfadia Y., Friedman R., Kadmon J., Selzer A., Nachliel E., Gutman M. (2007). Molecular dynamics simulations of palmitate entry into the hydrophobic pocket of the fatty acid binding protein. FEBS Lett.

[42] Klimovich P.V., Shirts M.R., Mobley D.L. (2015). Guidelines for the analysis of free energy calculations. J Comput Aided Mol Des.

[43] Sulimov A., Zheltkov D., Oferkin I., Kutov D., Tyrtyshnikov E., Sulimov V. (2016). Novel gridless program SOL-P for flexible ligand docking with moveable protein atoms. 21st EuroQSAR where molecular simulations meet drug discovery, September 4–8, 2016. Aptuit Conference Center, Verona Italy, abstract book, OC15.

[44] Halgren T.A. (1996). Merck molecular force field. I. Basis, form, scope, parameterization and performance of MMFF94. J Comput Chem.

[45] Katkova E.V., Onufriev A.V., Aguilar B., Sulimov V.B. (March 2017). Accuracy comparison of several common implicit solvent models and their implementations in the context of protein-ligand binding. J Mol Graph Model.

[46] Cornell W.D., Cieplak P., Bayly C.I., Gould I.R., Merz K.M., Ferguson D.M. (1995). A second generation force field for the simulation of proteins, nucleic acids, and organic molecules. J Am Chem Soc.

[47] Wang J., Wolf R.M., Caldwell J.W., Kollman P.A., Case D.A. (2004). Development and testing of a general amber force field. J Comput Chem.

[48] Jorgensen W.L., Maxwell D.S., Tirado-Rives J. (1996). Development and testing of the OPLS all-atom force field on conformational energetics and properties of organic liquids. J Am Chem Soc.

[49] Vanommeslaeghe K., Hatcher E., Acharya C., Kundu S., Zhong S., Shim J. (2010). CHARMM general force field: a force field for drug-like molecules compatible with the CHARMM all-atom additive biological force fields. J Comput Chem.

[50] Sinauridze E.I., Romanov A.N., Gribkova I.V., Kondakova O.A., Surov S.S., Gorbatenko A.S. (2011). New synthetic thrombin inhibitors: molecular design and experimental verification. PLoS One.

[51] Sulimov V.B., Katkova E.V., Oferkin I.V., Sulimov A.V., Romanov A.N., Roschin A.I. (2014). Application of molecular modeling to Urokinase inhibitors development. Biomed Res Int.

[52] Sulimov V.B., Gribkova I.V., Kochugaeva M.P., Katkova E.V., Sulimov A.V., Kutov D.C. (2015). Application of molecular modeling to development of new factor Xa inhibitors. https://www.hindawi.com/journals/bmri/2015/120802/.

[53] Oseledets I.V., Tyrtyshnikov E.E. (2009). Breaking the curse of dimensionality, or how to use SVD in many dimensions. SIAM J Sci Comput.

[54] Bader B., Kolda T. (2009). Tensor decompositions and applications. SIAM Rev.

[55] Oseledets I.V., Tyrtyshnikov E.E. (2010). TT-Cross approximation for multidimensional arrays. Linear Algebra Appl.

[56] Oseledets I.V. (2011). Tensor-train decomposition. SIAM J Sci Comput.

[57] Goreinov S.A., Tyrtyshnikov E.E., Zamarashkin N.L. (1995). Pseudo-skeleton approximations of matrices.

[58] Goreinov S.A., Tyrtyshnikov E.E., Zamarashkin N.L. (1997). A theory of pseudo-skeleton approximations. Linear Algebra Appl.

[59] Tyrtyshnikov E.E. (2000). Incomplete cross approximation in the mosaic-skeleton method. Comput Secur.

[60] Goreinov S.A., Tyrtyshnikov E.E. (2001). The maximal-volume concept in approximation by low-rank matrices. Contemp Math.

[61] Goreinov S.A., Oseledets I.V., Savostyanov D.V., Tyrtyshnikov E.E., Zamarashkin N.L. (2008). How to find a good submatrix. Research report 8–10.

[62] Zheltkov D.A., Tyrtyshnikov E.E. (2015). Parallel implementation of matrix cross method. Vychislitelnye Metody Programmirovanie (Numer Methods Program).

[63] Cole J.C., Nissink J.W.M., Taylor R., Alvarez J., Shoichet B. (2005). Protein-ligand docking and virtual screening with GOLD. Virtual screening in drug discovery.

[64] Berman H.M., Westbrook J., Feng Z., Gilliland G., Bhat T.N., Weissig H. (2000). The protein data bank. Nucleic Acids Res.

[65] Nelder J.A., Mead R. (1965). A simplex method for function minimization. Comput J.

[66] Rowan T. (1990). Functional stability analysis of numerical algorithms.

[67] Johnson Steven G. The NLopt nonlinear-optimization package. http://ab-initio.mit.edu/nlopt.

[68] Byrd R.H., Lu P., Nocedal J., Zhu C. (1995). A limited memory algorithm for bound constrained optimization. SIAM J Sci Comput.

[69] Zhu C., Byrd R.H., Lu P., Nocedal J. (1997). Algorithm 778: L-BFGS-B: Fortran subroutines for large-scale bound-constrained optimization. ACM Trans Math Softw.

[70] Press W.H., Teukolsky S.A., Vetterling W.T., Flannery B.P. (2007). Section 22.4 Cyclic redundancy and other checksums. Numerical recipes: the art of scientific computing.

[71] Donald E.K. (1997). The art of computer programming vol 1.

[72] Kuhn H.W. (1955). The Hungarian method for the assignment problem. Nav Res Logist Q.

[73] Sadovnichy V.A., Tikhonravov A.V., Voevodin V.V., Opanasenko V. (2013). “Lomonosov”: supercomputing at Moscow State University. Contemporary high performance computing: from petascale toward exascale.

[74] Avogadro: an open-source molecular builder and visualization tool. Version 1. XX. http://avogadro.cc/wiki/Main_Page.

